# Associations of Socioeconomic Status, Parental Smoking and Parental E-Cigarette Use with 10–11-Year-Old Children’s Perceptions of Tobacco Cigarettes and E-Cigarettes: Cross Sectional Analysis of the CHETS Wales 3 Survey

**DOI:** 10.3390/ijerph17030683

**Published:** 2020-01-21

**Authors:** Graham F. Moore, Lianna Angel, Linsay Gray, Lauren Copeland, Jordan Van Godwin, Jeremy Segrott, Britt Hallingberg

**Affiliations:** 1 DECIPHer, School of Social Sciences, Cardiff University, Wales CF10 3BD, UK; AngelL@cardiff.ac.uk (L.A.); CopelandLC@cardiff.ac.uk (L.C.); vangodwinj1@cardiff.ac.uk (J.V.G.); 2 SPECTRUM Consortium, UK; 3 MRC/CSO Social and Public Health Sciences Unit, University of Glasgow, Scotland G2 3AX, UK; linsay.gray@glasgow.ac.uk; 4 DECIPHer, Centre for Trials Research, Cardiff University, Wales CF14 4YS, UK; SegrottJ@cardiff.ac.uk; 5 Cardiff School of Sport and Health Sciences, Cardiff Metropolitan University, Wales CF5 2YB, UK; BHallingberg@cardiffmet.ac.uk

**Keywords:** e-cigarettes, tobacco, smoking, children, parents

## Abstract

Background: This study examines primary schoolchildren’s perceptions of e-cigarettes and tobacco cigarettes, and associations with parental smoking, vaping and socioeconomic status. Methods: Survey of 2218 10–11-year-old children in 73 schools in Wales. Results: Overall, 36% reported that a parent figure smoked compared to 21% for vaping, with parental smoking lower in affluent families (OR = 0.72; 95% CI = 0.68 to 0.76). Overall, 1% had tried a cigarette, while 5% had tried an e-cigarette. Most said they would not smoke or vape in 2 years’ time; susceptibility to vaping (20%) was higher than smoking (12%). Exposure to and perceptions of tobacco cigarettes were more positive for children of smokers. Having a parent who vaped was associated with exposure to and positive perceptions of e-cigarettes, but not smoking. Most children perceived e-cigarettes as used by adults to stop smoking (64%). Susceptibility to smoking (OR = 0.57; 95% CI = 0.41 to 0.79) and vaping (OR = 0.78; 95% CI = 0.62 to 0.99) were lower among children who perceived e-cigarettes as cessation aids. Conclusions: Parental smoking continues to be concentrated in poorer families. This study provides no evidence that parental vaping in the absence of smoking is associated with more positive perceptions of tobacco cigarettes. Communicating to children the role of e-cigarettes as cessation devices for smokers may help to limit their appeal to young people.

## 1. Introduction

Having a parent figure who smokes is a well-established risk factor for smoking initiation in childhood and adolescence; a meta-analysis of 58 studies, for example, found that the odds of smoking uptake doubled where a parent figure smoked [1]. Smoking rates have declined substantially in recent years [2], with progressively comprehensive tobacco control strategies acting to de-normalise smoking [3]. However, many parents continue to smoke, with parental smoking particularly common among less affluent families [4]. Given the strong intergenerational transmission of both socioeconomic status and smoking [5], smoking remains a major contributor to the intergenerational reproduction of health inequalities [6].

E-cigarettes emerged in the United Kingdom (UK) around 2011 and have been widely used by adults to quit smoking in the UK [7]. For example, data from Wales show that adult smoking reached an all-time low in 2019, while e-cigarettes had become the most commonly used method to support quit attempts [8]. Data from England suggest that e-cigarettes may support between 50,000 and 70,000 successful quit attempts per year [9]. In a recent randomised trial, e-cigarettes were almost twice as effective in supporting cessation as other nicotine replacement therapies, where both arms also received behavioural support [10]. 

Harms remain disputed, and perspectives differ among academics [11], policymakers, health professionals and the public on how to manage this uncertainty [12,13]. At the time of submission for example, an outbreak of pulmonary conditions and its links to use of e-cigarettes are the subject of widespread international debate [14]. While this situation is evolving, the latest evidence indicates that these incidents are in large part related to chemicals including tetrahydrocannabinol and vitamin E acetate which are not present within products sold legally in the UK [15,16]. While *absolute* harms of e-cigarettes continue to be debated, and are unlikely to be fully known for some decades, a recent randomised trial provides perhaps the strongest evidence to date of reduced *relative* harm on switching from smoking to e-cigarette use, finding improved cardiovascular function within a month [17]. Hence, given growing UK evidence of positive impacts on cessation [8,9,18,19], and good reason to believe e-cigarettes (at least those which are sold legally in the UK) are less harmful than smoking, many endorse their use within harm reduction strategies [20]. While not uncontroversial [21], Public Health England for example advocate use of e-cigarettes for smokers as a means of quitting smoking [22]. For parents, if e-cigarettes help them to give up smoking, and are less harmful than smoking, this may offer a means of reducing their own chronic disease risk. Further, given the established links between parental smoking and young people’s uptake of smoking, by giving up, parents might also reduce the risk of transmission of smoking behaviours to their children.

However, key concerns driving debates around the regulation of e-cigarettes have been their perceived role as a ‘gateway’ into smoking for children and adolescents [23,24], and perceptions that they might renormalise smoking [25]. Gateway and renormalisation theories are often conflated in public discourse [26]. However, notions of e-cigarettes as a gateway to smoking suggest that young people who would not otherwise have used tobacco cigarettes, but who experiment with nicotine via e-cigarettes, will be at increased risk of going on to use tobacco cigarettes [23,24]. Proponents of renormalisation focus on ideas that growing visibility of e-cigarette use may increase the extent to which the ‘similar’ behaviour of smoking is seen as normal by children and adolescents [25]. These theories have been influential in shaping international policy debates and decisions. For example, the EU Tobacco Products Directive (TPD) justifies regulation on the basis that e-cigarettes act as a gateway to nicotine addiction, and renormalise smoking through mimicking its action [27]. In Wales, moves to extend smoke-free legislation to restrict use of e-cigarettes in public places [28] were justified on the basis that e-cigarettes would renormalise smoking. International regulatory frameworks have become increasingly divergent, with India for example recently announcing a ban on sales of e-cigarettes, due to concerns regarding a potential youth epidemic in use [29]. Moves such as flavour bans have been debated widely in the US; advocates argue that such a ban would reduce appeal to children [30], while critics argue this would make adults more likely to choose tobacco cigarettes [31].

There is growing evidence that e-cigarette use among never smoking children and adolescents is associated with later smoking [32], including within the UK [33]. However, as the authors acknowledge [33], much of this observed association may reflect residual confounding by shared risk factors, rather than one causing the other. For example, as most adult e-cigarette use is among adults who smoke or have recently given up [7,8,9,18,19], it logically follows that most children whose parents use e-cigarettes will come from families where they are currently, or have recently also been, exposed to tobacco cigarettes. Analyses of survey data from three UK nations found that experimentation with tobacco cigarettes declined during the emergence of e-cigarettes, while the decline in perceptions of acceptability of smoking accelerated [2]. Hence, this provides no evidence that smoking was becoming renormalised during this time and suggests that any gateway effects were not having a population level impact on smoking rates. Similar findings have been reported among older adolescents and young adults in the United States (US) [34]. However, newer generation high nicotine devices are gaining popularity among North American youth, while there is some evidence that smoking has also increased [35]. These products have not entered UK markets in the same form due to differences in regulatory frameworks, while regular use of e-cigarettes has remained comparatively low among UK youth [36].

While these issues have received significant and growing international attention in adolescence when most smoking initiation occurs, fewer studies have focused on perceptions of tobacco cigarettes and e-cigarettes earlier in childhood. An early study with 6–10-year-old children in New Zealand [37] found little awareness of e-cigarettes and a tendency not to differentiate between them and smoking. However, as New Zealand is a context of strict regulation of e-cigarettes and interviews were conducted early in their emergence, transferability to the present day in the UK is questionable. In countries such as the UK, today’s primary school-aged children are the first cohort to have been born following smoke-free legislation, and to have grown up in an environment of substantially reduced tobacco smoking in the presence of children [4,38,39,40]. They are also the first generation for whom e-cigarettes have been a significant presence throughout their lives [41]. Understanding the formation of smoking and e-cigarette perceptions in this group is therefore vital to inform contemporary policy approaches to preventing youth tobacco uptake.

Our previous survey of primary school-aged children in Wales found that two-thirds of never smoking children had heard of e-cigarettes, while 6% had tried an e-cigarette by 2014 [42]. More recent data from eight primary schools funded by Public Health Wales in 2017 found that children were generally aware of e-cigarettes and, contrasting to the New Zealand study, viewed e-cigarettes and tobacco cigarettes as different products [43]. E-cigarettes were seen as harmful, but less so than tobacco, while the most common reason cited for use of e-cigarettes was quitting smoking. Given that the re-normalisation hypothesis rests on assumptions that children see e-cigarettes and tobacco cigarettes as similar [44], emerging evidence that these are seen by children as different to tobacco cigarettes may indicate that exposure to parental use of e-cigarettes is unlikely to lead to more positive perceptions toward tobacco smoking. There remain, however, outstanding questions for stakeholders such as parents, health professionals, school staff and policymakers regarding whether parents’ use of e-cigarettes continues to communicate to children that smoking is normal, or conversely, becomes a social display of anti-smoking behaviour, communicating that smoking is undesirable [45].

This paper analyses data from a nationally representative sample of 10–11-year-old children conducted in Wales in 2019. It provides a unique cross-sectional overview of attitudes, behaviour and norms in relation to e-cigarette and tobacco use in a cohort of children growing up in the era of e-cigarettes and comprehensive tobacco control. It then assesses associations of socioeconomic status, parental smoking and parental vaping with these outcomes, including whether associations of parental vaping with perceptions of tobacco cigarettes remain or are fully attenuated, after adjustment for the co-occurrence of parental tobacco cigarette and e-cigarette use. Finally, we test the hypothesis that children who perceive e-cigarettes as devices to help adults quit smoking will be less likely to report susceptibility to smoking, and to perceive smoking as normal compared to children who perceive adult e-cigarette use as having other main purposes.

## 2. Materials and Methods

### 2.1. Sampling

All schools who participated in our previous nationally representative survey [42] were invited to participate in the 2019 Children’s Health E-cigarettes and Tobacco Study (CHETS) Wales 3 survey, which repeated many of the measures used in our earlier surveys and introduced a wider range of items in relation to e-cigarettes. Where schools declined or could not be contacted, another school was randomly selected from the same strata, defined by local authority and high/low free school meal (FSM) entitlement. Within each participating school, all Year 6 (aged 10–11 years) pupils were invited to complete the survey. In a small number of cases, schools taught some Year 5 (aged 9–10 years) students within the same class as Year 6 pupils, and, in these instances, all students within the class were included in the survey.

### 2.2. Consent and Data Collection

Consent was obtained in three stages. First, a signed agreement which outlined the data collection process was obtained from the head teacher of all participating schools. Second, parents were given the chance to opt their child out of the study by returning a freepost opt-out slip to the research team. A small number of parents returned the slip to the school and their children were opted out on the day. Third, pupils were given the option to not take part on the day, those who wished to take part completed an assent form which was read to them by a researcher to maximise understanding and answer any questions. Pupils were assured that participation was voluntary, and they could leave any question they did not want to answer or withdraw at any time. Study protocols were reviewed and approved by the Cardiff University School of Social Sciences Research Ethics Committee. Midway through the data collection period, one school referred the study to their local authority to assess data sharing and consent processes before agreeing to proceed. Recruitment and data collection were put on hold in this area while a response was awaited. The local government Data Protection Officer approved continuation without changes after reviewing study documentation. All researchers were provided with a data collection protocol and given training to maximise standardisation of data collection sweeps and attended the school at a time and date agreed with the head teacher. A summary was presented with a reminder that participation was voluntary, and all pupils completed the assent form before commencing the survey. Researchers remained in the room to answer queries while children completed the survey. The class teacher was asked to remain present, though not to intervene in the data collection unless asked to do so by a member of the research team.

### 2.3. Measures

#### 2.3.1. Demographics

Children were asked “are you a (i) boy, (ii) girl, (iii) prefer to self-describe, (iv) prefer not to say”. To measure affluence at the pupil level, we used items from the Family Affluence Scale [46] (FAS), which has been used in the WHO Health Behaviour in School-aged Children survey. This includes items on material affluences (e.g., bedroom occupancy, car and computer ownership, and bathrooms in the home) which were summed to form a total score, categorised into approximately equal thirds for descriptive analyses though included as a continuous variable in subsequent models. We excluded the item on family holidays typically used with secondary school pupils, due to confusion identified in our survey development and Public Involvement work (in which we asked a small number of children not in the study sample to read the questionnaire and provide feedback on their understandings of the questions within it), regarding what constituted a ‘holiday’, and poor correlation with school-level free school meal entitlement in previous rounds. When aggregated at the school level, the 5 item scale correlated highly with free school meal entitlement (r = 0.76).

#### 2.3.2. Parental Smoking and Vaping

In order to categorise children according to whether parent figures smoked, children were asked ‘Do any of the following people smoke?’ in relation to (i) father, (ii) mother, (iii) mother’s partner, (iv) father’s partner. A parent figure was classified as smoking if the child responded ‘smokes every day’ or ‘smokes sometimes’, with all other responses classified as non-smoking parent figures. Items were coded into a binary variable indicating having no parent figures who smoke, or at least one parent figure who smokes. Children were also asked using the same question format whether any parent figures used e-cigarettes. For questions on e-cigarettes, these were defined as devices used to inhale a vapour, sometimes called vaping, which may contain nicotine and are commonly flavoured, with pictures of common types of e-cigarettes provided.

#### 2.3.3. Exposure to Tobacco and E-Cigarettes in Shops

Children were asked whether, in the past month, they had seen tobacco in shops, and had seen e-cigarettes in shops, with response options of yes and no for each item.

#### 2.3.4. Child Ever Smoking, Awareness of E-Cigarettes, Ever E-Cigarette Use and Perceived Susceptibility to Future Smoking and Vaping

Children were asked whether they had ever smoked tobacco, with response options of yes or no. Children were also asked if they had heard of e-cigarettes (yes, no or I don’t know) with no and I don’t know responses combined to form a binary item, as well as whether they ever used an e-cigarette (yes once; yes more than once; never), with any use combined to indicate ever use. For both tobacco cigarettes and e-cigarettes, children were also asked how often they used these products at present (daily, at least once a week, less than once a week or I don’t, with daily or at least once week combined to indicate weekly use. As indicators of perceived smoking and vaping susceptibility, children were asked whether they thought they would (i) smoke or (ii) vape in 2 years’ time, with response options from ‘Definitely not’ to ‘Definitely yes’.

#### 2.3.5. Perceived Norms for Smoking and Vaping

Children were asked to rate perceived prevalence of smoking in Wales by indicating (i) how many children their age in Wales smoked, and (ii) how many adults in Wales smoked. Response options were ‘nearly all’, ‘about three-quarters’, ‘about half’, ‘about a quarter’, ‘hardly any’ and ‘I don’t know’. Questions for vaping repeated this same format.

#### 2.3.6. Perceived Reasons for Adults Using E-Cigarettes

Children were presented with a list and asked to select all which they thought were important reasons why adults used e-cigarettes, including (i) to stop smoking; (ii) to use where tobacco smoking is not allowed; (iii) because they are cheaper than tobacco or (iv) some other reason.

### 2.4. Statistical Analysis

We estimated frequencies and percentages overall and by sub-group for all key outcomes of interest, followed by odds ratios and 95% confidence intervals from logistic regression analyses, with standard errors adjusting for clustering at the school level in Stata 14.0. For each outcome, models are presented in a sequence including (i) FAS, (ii) FAS and parental smoking, (iii) FAS and parental vaping, (iv) FAS, parental smoking and vaping simultaneously enabling assessment of whether parental smoking and vaping associations with outcomes remain independent of one another. All models additionally adjust for sex and region of Wales. While parental smoking and parental e-cigarette use overlap substantially, collinearity was negligible (i.e., Variance Inflation Factors < 2), indicating no statistical problem with inclusion of both in the same model. Nevertheless, as a sensitivity analysis, we combined items on parental smoking and vaping into a single 4 category variable (including children with parents who (i) neither smoked nor vaped, (ii) smoked only, (iii) vaped only or (iv) both) and compared the latter three categories individually with the reference category (neither). This alternative analysis led to the same conclusions as our main analysis and hence we report only the analysis described above. We first examined associations with awareness of e-cigarettes and tobacco, comprising reports of whether children had heard of e-cigarettes, whether a parent figure smokes and vapes, and whether they had seen e-cigarettes and tobacco cigarettes in shops in the past month. We then examined associations with experimentation with tobacco and e-cigarettes and susceptibility to future use of tobacco and e-cigarettes. While frequencies present breakdowns of original raw items for future susceptibility on a 5-point Likert scale, for logistic regression analyses these variables were divided into two categories (i.e., ’definitely will not’ smoke in 2 years’ time vs. all other responses). We next assessed associations with four items relating to perceived smoking norms which were the percentage of children reporting that: (i) ‘most adults smoke’ (i.e., selecting a response > 50% when asked to estimate the prevalence of adult smoking in Wales), (ii) ‘most adults vape’, (iii) the percentage giving any response other than ‘hardly any’ when asked how many children in Wales smoke, and (iv) the percentage giving any response other than ‘hardly any’ when asked how many children in Wales vape. We then examined associations with children’s (non-mutually exclusive) perceptions of why adults use e-cigarettes including models for (i) to stop smoking, (ii) so that they can use them where cigarettes aren’t allowed (iii) because they are cheaper than cigarettes or (iv) other. Finally, we examined how children’s perceived reasons for adults’ use of e-cigarettes were associated with perceptions of smoking and vaping norms and susceptibility using multivariable logistic regression analyses adjusted for FAS, sex and parental smoking and vaping. No weights were used in previous CHETS surveys, although due to lower school-level response rates in the 2019 survey, we considered their use for this survey. While weights are commonly applied in school-based surveys to ensure representativeness by age and sex, the analysis focused on a single year group and included an even split of boys and girls. Further, we considered weighting by school level Free School Meal entitlement and region. However, the sample showed minimal departures from population estimates of socioeconomic status or regional distribution of participating pupils across Wales. Hence, we chose not to apply weights. For the vast majority of items, missing data were ≤ 5%, and hence no imputation was used. Successively adjusted models were constructed using both list-wise deletion and complete cases for each dependent variable. As findings were consistent across approaches, only those for the former are presented.

## 3. Results

### 3.1. Response Rates and Sample Description

In total, 186 primary schools were invited to take part. Despite multiple contact attempts 36 schools did not respond, 73 schools declined, and 77 agreed to take part in principle, with four dropping out due to time/scheduling constraints. Overall, 73 schools took part in the study; a school-level response rate of 39.3%. Of 2514 pupils within sampled classes, 2218 (88.2%) took part in the survey, with 53 (2.1%) opted out by parents and 58 (2.3%) declining to participate, but most non-participation was due to absence on the day of the data collection visit (n = 185; 7.4%). Sample characteristics are described in Table 1.

### 3.2. Perceptions of, and Exposure to, Tobacco and E-Cigarettes in 2019

Table 2 presents frequencies and percentages of children reporting awareness of, exposure to and a range of perceptions of, e-cigarettes and tobacco overall, by parental smoking, parental vaping and family affluence level. For each of these associations odds ratios and 95% confidence intervals are presented in Table 3.

### 3.3. Awareness of E-Cigarettes, Parental Smoking and Vaping and Exposure to Tobacco and E-Cigarettes in Shops

As indicated in Table 2, the vast majority (84.0%) of children had heard of e-cigarettes, while awareness was particularly high among children of smokers (88.4%) and children of vapers (92.6%). Reports of parental smoking (and, to a lesser extent, vaping) were concentrated among children from poorer backgrounds, with 50.4% children from the poorest third of the distribution reporting that one or more parent figures smoked, compared to 18.7% of those from the most affluent group. Parental smoking and parental vaping overlapped substantially; most children who reported that a parent figure used e-cigarettes reported that a parent figure also smoked (68.8%). Most children (70.0%) recalled seeing tobacco cigarettes in shops in the past month, with about half as many (38.0%) citing recall of e-cigarettes in shops. Seeing tobacco and e-cigarettes in shops were both higher for children of smokers and children of vapers.

Table 3 indicates that awareness of e-cigarettes was significantly higher among children with a parent figure who smoked and children with a parent figure who vaped, with associations for parental smoking and vaping remaining significant in mutually adjusted models. There was no significant association between family affluence and awareness of e-cigarettes, although in models adjusting for parental smoking and vaping simultaneously, a greater awareness of e-cigarettes in children from more affluent families neared significance (*p* = 0.06). Being from a poorer family, and parental vaping were independently associated with a greater likelihood of having a parent figure who smoked. Being from a poorer family was associated with increased parental vaping, but this association became non-significant after adjustment for parental smoking. Having seen tobacco in shops was associated with both parental smoking and vaping where considered separately, but where mutually adjusted, only parental smoking independently predicted exposure. There were no significant associations between family affluence and having seen tobacco in shops or having seen e-cigarettes in shops. Both parental smoking and vaping were significantly associated with having seen e-cigarettes in shops in separate and mutually adjusted models.

### 3.4. Experimentation and Perceived Future Susceptibility to Smoking and Vaping

Table 2 indicates that fewer than 1% (0.9%) of 10-year-old children reported that they had tried a cigarette; the vast majority of those who had tried smoking reported having parents who smoke. Approximately 5% (4.9%) of children reported having tried an e-cigarette, with ever use higher for children of smokers and vapers. In most cases, children reported using an e-cigarette only once (3.7% once vs. 1.2% more than once), while few experimental users of either product reported current weekly use (<=5 cases). Hence weekly use was not analysed further. Overall, most children said they will definitely not smoke and will definitely not vape in 2 years’ time. Almost 9 in 10 (87.9%) children said they will definitely not smoke in 2 years’ time, while very few (0.6%) said that they definitely will. However, percentages saying ‘probably not’ or ‘maybe’ were higher for children of smokers (9.4% and 6.3% respectively) compared to children of non-smokers (6.4% and 1.8% respectively). More children provided a response other than ‘definitely not’ for vaping (20.2%) than for smoking (12.1%). Having a parent figure who smokes was significantly and robustly associated with all smoking, vaping and future susceptibility outcomes (Table 3). Parental vaping was significantly associated with all outcomes, except ever smoking, in models which did not adjust for parental smoking. However, after adjustment for parental smoking, parental vaping remained significantly associated only with vaping susceptibility. In no model was smoking or vaping experimentation or susceptibility significantly associated with family affluence.

### 3.5. Perceived Norms for Vaping and Smoking

As indicated in Table 2, approximately 1 in 3 (31.4%) children reported a perception that most adults in Wales smoke, with the perception most common in children of smokers and, to a lesser extent, children of vapers and those from poorer families. Fewer children (24.5%) reported that vaping was something most adults do. Around a quarter of children selected an option other than ‘hardly any’ when asked how many children their age smoked (26.9%) or vaped (27.9%). In models considering parental vaping and smoking separately (Table 3), both predicted a higher tendency to perceive smoking and vaping as normal adult behaviours. However, where mutually adjusted, parental smoking was associated with all normative items, whereas parental vaping was associated with perceived norms in relation to vaping, but not smoking. Family affluence was associated with perceived norms for smoking and vaping in unadjusted models, but not in models adjusted for parental smoking.

### 3.6. Perceived Reasons for Adults’ Use of E-Cigarettes

As indicated in Table 2, most (64.1%) children who reported awareness of e-cigarettes perceived them as being used by adults as a way of quitting smoking, particularly children of smokers and children of vapers, and among children from less affluent backgrounds. That e-cigarettes can be used where smoking is banned, or because they are cheaper than cigarettes, were cited by fewer children as perceived motivators of use by adults. These latter responses were less common among children of smokers and children of vapers, as well as children from less affluent backgrounds. In logistic regression analyses (Table 3), both parental smoking and vaping were significantly and robustly associated with perceiving e-cigarettes as a stop smoking device, with significant differences in family affluence in models prior to adjustment for parental smoking and vaping attenuated after adjustment. In models including parental smoking and vaping separately, both were associated with a lower likelihood of perceiving e-cigarettes to be used by adults to use nicotine in places where smoking was not allowed or because they are cheaper than smoking; only parental vaping remained significantly associated with both items following mutual adjustment.

### 3.7. Association of Perceived Reasons for Adults’ Use of E-Cigarettes with Smoking and Vaping Norms and Susceptibility

Table 4 presents multivariable logistic regression models examining associations of children’s (non-mutually exclusive) perceptions of why adults use e-cigarettes with perceived smoking norms, and susceptibility for future smoking and vaping (among children who had heard of e-cigarettes). A borderline negative association was observed between perceiving e-cigarettes as being used for smoking cessation and susceptibility for future vaping. A stronger negative association was observed for smoking susceptibility, with perceiving e-cigarettes as being used to help adults stop smoking associated with significantly lower reported smoking susceptibility. Children who reported that adults used e-cigarettes because they were cheaper than tobacco were more likely to perceive smoking as common among other children, though this reason was not significantly associated with other outcomes.

## 4. Discussion

While smoking rates have declined at the population level, parental smoking continues to be highly concentrated in more deprived families [4,5]. In the present study, half of children from the poorest families reported that a parent figure smoked, three times as many as for children from the most affluent families. Children with a parent who smoked were more likely to report susceptibility to future smoking, to be exposed to tobacco in shops and to view smoking among adults and children as a normal behaviour. There was little evidence of socioeconomic patterning in most childhood perceptions of tobacco or e-cigarettes. However, children from poorer families were more likely to perceive smoking as normal, with this association fully attenuated once parental smoking was accounted for. These findings add to a large existing evidence base which positions parental smoking as a major risk factor for smoking initiation [1], and a continued contributor to the intergenerational reproduction of inequalities [6].

This paper is one of the first to contribute to our understanding of perceptions of tobacco and e-cigarettes among the first generation of children raised after introduction of smoke-free legislation, and alongside the co-occurring emergence of e-cigarettes. Concern has been expressed that e-cigarettes might act as a gateway into tobacco use, or renormalise smoking for young people. While this debate has received much attention in research focused on adolescents [47,48,49], few studies have focused on these issues among young children [42]. In 2019, while awareness of e-cigarettes was high, children remained more likely to be exposed to tobacco than to e-cigarettes; almost twice as many reported that a parent smoked or that they had seen tobacco cigarettes in shops in the past month by comparison with e-cigarettes. Children were more likely to perceive that most adults smoked than to perceive that most adults’ vaped, although perceived prevalence of smoking and vaping among children were similar. While exposure to tobacco cigarettes remained higher than for e-cigarettes, some perceptions of e-cigarettes appeared more positive, with more children reporting susceptibility to vaping in 2 years’ time than smoking. Childhood experimentation with e-cigarettes was substantially higher than with tobacco cigarettes, though both were marginally less prevalent in 2019 than in our previous survey in 2014 [42]. Among children who had tried an e-cigarette, the vast majority had done so only once, while regular use of both products was very rare.

We found substantial overlap between parental smoking and vaping; children with a parent figure who vaped had greater odds of having a parent who smoked, and vice versa. While some previous research finds greater use of e-cigarettes among more advantaged socioeconomic groups [50], or limited evidence of use by groups in which smoking is commonly concentrated [51], reports of parental e-cigarette use were higher among children from more deprived families in our sample, although these socioeconomic differences were attenuated to non-significance on adjustment for the higher rates of parental smoking in poorer families. Where parental smoking was not adjusted for, having a parent figure who vaped was associated with almost all measured outcomes, including greater exposure to tobacco cigarettes and e-cigarettes in shops, increased susceptibility to vaping and smoking, and a greater likelihood of perceiving both vaping and smoking as normal. Once parental smoking was included within these models, associations of parental vaping with perceptions of smoking were attenuated to non-significance. Only perceptions of vaping norms and susceptibility, and exposure to e-cigarettes themselves remained significantly associated with parental vaping after adjustment for parental smoking. Notably, children’s own experimentation with both tobacco cigarettes and e-cigarettes were more strongly associated with parental smoking than with parental vaping. Child experimentation with tobacco occurred almost exclusively among children of smokers, while associations of parental vaping with experimentation with e-cigarettes became non-significant after adjustment for parental smoking. Given that e-cigarettes are used primarily by smokers and ex-smokers, most children with a parent figure who vaped but didn’t smoke were likely to be children of recent ex-smokers [7]. Hence, the finding that parental vaping in the absence of continued smoking is not associated with more positive perceptions of tobacco cigarettes offers some re-assurance that if parents give up smoking via e-cigarettes, there is no evidence that continued use of a nicotine inhaling device which mimics the act of smoking will serve to maintain positive perceptions of smoking among their children. Nevertheless, as a large proportion of children with parents who used e-cigarettes did continue to have parents who smoked, continued smoking alongside e-cigarette use likely maintains an elevated risk of childhood smoking initiation. Further, our study does provide some evidence that parental e-cigarette use is associated with more positive norms and susceptibility for vaping itself, independent of the association of parental e-cigarette use with parental smoking.

Building on previous work in the Welsh primary school context [43], we found that among children who had heard of e-cigarettes, a majority perceived these as devices used by adults to quit smoking. Notably, children with parents who smoked or used e-cigarettes, who were perhaps more likely to interact directly with adults who used e-cigarettes in their daily lives, were substantially more likely to perceive e-cigarettes as stop smoking devices. Hence, adult e-cigarette use was perhaps explained and interpreted as a social display of an attempt to quit smoking [45]. Children’s reported future smoking and vaping susceptibility were both significantly lower among those for whom e-cigarettes were perceived as devices to help adults stop smoking. In contrast to recent qualitative research, which found that poorer adults switched from tobacco cigarettes to e-cigarettes primarily because they were cheaper [52], we found that children from more affluent families were more likely to cite this as a reason for use. However, parents may communicate to their children that they are using e-cigarettes to stop smoking, without in turn communicating that this is due to issues of cost.

A major strength of this study is the large, nationally representative sample of children in Wales. However, limitations include reliance of self-report measures of child and parent behaviour, from the perspective solely of the child, and the cross-sectional nature of the study, which is unable to establish cause and effect relationships. Separating effects of exposure to tobacco cigarettes from exposure to e-cigarettes remains challenging due to their continued overlap. Hence, longitudinal data, with large sample sizes, are needed to further understand the impacts of e-cigarettes on the intergenerational transmission of smoking.

## 5. Conclusions

Nevertheless, the study has important implications for policy, practice and future research. A substantial proportion of children continue to have parent figures who smoke, and parental smoking remains concentrated in more deprived families. Hence, supporting parents to quit via interventions which are effective for less affluent parents remains vital in disrupting the intergenerational transmission of smoking and health inequalities. E-cigarettes have emerged as a popular means for UK adults to stop smoking and appear to be widely used by parents throughout the socioeconomic distribution in Wales. While these may offer substantial harm reduction potential for parents who quit smoking using them, attention to their impacts on children and young people remains vital. Our findings are consistent with a conclusion that the tendency for more positive perceptions of tobacco cigarettes among children with parent figures who use e-cigarettes is explained by the fact that most of these children also have parent figures who use tobacco cigarettes. Hence, our study provides no evidence to support a view that if parent figures fully switch from tobacco smoking to e-cigarettes, the use of devices which ‘mimic’ the act of smoking will maintain positive perceptions of smoking among their children. This adds to prior evidence that, in the UK at least, the presence of e-cigarettes in the lives of children who have grown up in a context of comprehensive tobacco control, has not led them to perceive tobacco cigarettes more positively. Childhood experimentation with e-cigarettes, while showing no growth on previous estimates, ought to be prevented. The finding that where children perceive e-cigarettes as things adults use to quit smoking this is associated with lower smoking and vaping susceptibility, suggests that effective communication by families, schools and policymakers regarding the role of these devices as smoking cessation aids may have an important role to play in limiting their appeal to children. Development and evaluation of acceptable messaging to educate children about the role of e-cigarettes is a priority for future research. Ongoing monitoring of the impacts of e-cigarettes on children and young people remains vital as the products and the landscape in which they are sold, used and regulated continue to evolve.

## Figures and Tables

**Table 1:** Sample description and comparison with population estimates.

	CHETS 3 Survey Population	All-Wales Population Estimates
	N (%) Unless Otherwise Indicated	
Total	2218	-
School-level free school meal entitlement	16.8 (10.9) *	16.1 (11.3) *
Region of Wales		
North	461 (20.8)	18.0%
South	1504 (67.8)	65.8%
West	108 (4.9)	5.9%
Mid	145 (6.5)	10.3%
Gender		
Male	1119 (50.7)	-
Female	1073 (48.6)	-
Prefer to self-describe/prefer not to say	17 (0.8)	-
Lives with		
Both parents	1509 (68.0)	-
Step family	206 (9.3)	-
Single mum	356 (16.1)	-
Single dad	29 (1.3)	-
Grandparents	29 (1.3)	-
Care/foster home	12 (0.5)	-
Other/missing	77 (3.5)	-

* Mean percentage (and standard deviation).

**Table 2:** Frequency (and percentage) for children’s perceptions of and exposure to tobacco and e-cigarettes by parental smoking and vaping and family affluence.

		Family Affluence			Parent Figure Who Smokes		Parent Figure Who Vapes		Total
		Low	Medium	High	No	Yes	No	Yes	
Awareness									
Heard of e-cigarettes		636 (84.6)	701 (82.8)	473 (86.0)	1092 (82.0)	669 (88.4)	1362 (82.1)	403 (92.6)	1847 (84.0)
Parent figure who smokes		365 (50.4)	279 (34.6)	100 (18.7)			432 (26.8)	293 (68.8)	765 (36.3)
Parent figure who vapes		189 (26.3)	167 (20.7)	72 (13.4)	133 (10.1)	293 (40.4)			437 (20.8)
Seen in shops (tobacco)		531 (74.2)	544 (67.1)	367 (69.4)	823 (64.0)	582 (81.6)	1096 (68.8)	315 (76.6)	1470 (70.0)
Seen in shops (e-cigarettes)		273 (40.3)	285 (36.5)	198 (38.5)	432 (34.6)	306 (45.3)	547 (35.6)	193 (49.4)	765 (38.0)
Experimentation and susceptibility									
Ever smoked		10 (1.3)	5 (0.6)	*	*	15 (2.0)	11 (0.7)	8 (1.8)	20 (0.9)
Ever used an e-cigarette		43 (5.8)	35 (4.2)	26 (4.7)	36 (2.7)	68 (9.0)	62 (3.8)	39 (9.0)	108 (4.9)
Will you smoke in 2 years?	Definitely yes	7 (1.0)	*	*	*	7 (1.0)	6 (0.4)	5 (1.2)	12 (0.6)
	Probably yes	8 (1.1)	*	10 (1.9)	11 (0.8)	8 (1.1)	10 (0.6)	5 (1.2)	15 (0.7)
	Maybe, maybe not	34 (4.7)	24 (2.9)	16 (3.0)	23 (1.8)	46 (6.3)	47 (2.9)	23 (5.5)	75 (3.5)
	Probably not	52 (7.2)	70 (8.4)	34 (6.3)	83 (6.4)	69 (9.4)	117 (7.2)	36 (8.7)	158 (7.4)
	Definitely not	624 (86.1)	736 (88.7)	479 (88.9)	1191 (91.1)	601 (82.2)	1448 (88.9)	347 (83.4)	1881 (87.9)
Will you vape in 2 years?	Definitely yes	10 (1.4)	*	5 (0.9)	*	14 (1.9)	10 (0.6)	7 (1.7)	20 (0.9)
	Probably yes	18 (2.5)	16 (2.0)	11 (2.0)	17 (1.3)	28 (3.9)	22 (1.4)	18 (4.3)	42 (2.0)
	Maybe, maybe not	51 (7.2)	48 (5.8)	32 (5.9)	54 (4.2)	73 (10.1)	80 (5.0)	47 (11.3)	135 (6.4)
	Probably not	79 (11.1)	92 (11.1)	56 (10.4)	120 (9.2)	102 (14.1)	147 (9.1)	75 (18.1)	232 (10.9)
	Definitely not	551 (77.7)	671 (81.1)	436 (80.7)	1108 (85.3)	505 (69.9)	1356 (84.0)	268 (64.6)	1694 (79.8)
Perceived smoking norms									
Most adults smoke		267 (36.1)	246 (29.5)	155 (28.5)	327 (24.9)	323 (43.6)	472 (28.8)	170 (40.3)	678 (31.4)
Most adults vape		195 (27.6)	187 (23.2)	122 (22.9)	263 (20.6)	230 (32.1)	341 (21.5)	144 (35.0)	513 (24.5)
More than hardly any children my age smoke		203 (28.2)	222 (26.8)	138 (25.7)	318 (24.5)	230 (31.6)	412 (25.5)	137 (32.7)	573 (26.9)
More than hardly any children my age vape		207 (29.5)	220 (27.2)	142 (27.0)	321 (25.1)	237 (33.6)	404 (25.5)	152 (37.0)	581 (27.9)
Why adults use e-cigarettes **									
To stop smoking		424 (66.8)	462 (65.9)	278 (58.9)	639 (58.6)	499 (74.7)	798 (58.6)	337 (83.8)	1183 (64.1)
To use where smoking is not allowed		112 (17.6)	169 (24.1)	113 (23.9)	276 (25.3)	108 (16.1)	342 (25.1)	44 (10.9)	399 (21.6)
Cheaper than smoking		108 (17.0)	119 (17.0)	108 (22.9)	228 (20.9)	100 (15.0)	281 (20.7)	43 (10.7)	340 (18.4)

* Cell counts <5 cases are suppressed (for ordinal items ns for suppressed category combined with next); ** limited to children who reported awareness of e-cigarettes; total sample sizes differ marginally due to missing data.

**Table 3:** Logistic regression analyses examining associations of family affluence and parental smoking/vaping with children’s smoking and vaping behaviours and intentions.

		Model 1 FAS Only	Model 2 FAS and Parental Smoking	Model 3 FAS and Parental Vaping	Model 4 FAS and Parental Smoking and Parental Vaping
Awareness					
Heard of e-cigarettes (n = 1986)	FAS	1.02 (0.94 to 1.11)	1.07 (0.99 to 1.17)	1.05 (0.97 to 1.14)	1.09 (1.00 to 1.18)
	Parent figure smokes	-	**1.88 (1.39 to 2.54)**	-	**1.55 (1.13 to 2.14)**
	Parent figure vapes	-		**3.16 (2.10 to 4.73)**	**2.62 (1.76 to 3.90)**
Parent figure who smokes (n = 1997)	FAS	**0.72 (0.68 to 0.76)**	-	**0.73 (0.69 to 0.78)**	-
	Parent figure vapes	-	-	**5.67 (4.35 to 7.39)**	-
Parent figure who vapes (n = 1997)	FAS	**0.85 (0.80 to 0.90)**	0.96 (0.90 to 1.02)	-	-
	Parent figure smokes	-	**5.71 (4.37 to 7.44)**	-	-
Seen in shops (tobacco) (n = 1906)	FAS	0.94 (0.89 to 1.00)	1.00 (0.94 to 1.07)	0.95 (0.89 to 1.01)	1.00 (0.93 to 1.06)
	Parent figure smokes	-	**2.69 (2.12 to 3.40)**	-	**2.65 (1.98 to 3.56)**
	Parent figure vapes	-	-	**1.50 (1.15 to 1.95)**	1.04 (0.77 to 1.40)
Seen in shops (e-cigarettes) (n = 1842)	FAS	0.99 (0.93 to 1.05)	1.03 (0.97 to 1.10)	1.01 (0.95 to 1.07)	1.03 (0.97 to 1.10)
	Parent figure smokes	-	**1.70 (1.43 to 2.01)**	-	**1.52 (1.26 to 1.82)**
	Parent figure vapes	-	-	**1.83 (1.48 to 2.26)**	**1.60 (1.29 to 1.98)**
Experimentation and susceptibility					
Ever smoked (n = 1905)	FAS	0.76 (0.57 to 1.02)	0.85 (0.62 to 1.17)	0.77 (0.56 to 1.05)	0.84 (0.60 to 1.16)
	Parent figure smokes		**5.58 (1.83 to 16.94)**		**4.99 (1.56 to 15.97)**
	Parent figure vapes			2.48 (0.98 to 6.27)	1.53 (0.59 to 3.96)
Ever used an e-cigarette (n = 1977)	FAS	0.95 (0.85 to 1.06)	1.05 (0.93 to 1.19)	0.99 (0.87 to 1.12)	1.06 (0.93 to 1.21)
	Parent figure smokes		**3.58 (2.23 to 5.74)**		**3.11 (1.90 to 5.10)**
	Parent figure vapes			**2.41 (1.45 to 3.98)**	1.51 (0.90 to 2.52)
Smoking susceptibility (n = 1942)	FAS	0.93 (0.86 to 1.01)	0.97 (0.90 to 1.05)	0.93 (0.86 to 1.01)	0.97 (0.89 to 1.05)
	Parent figure smokes		**2.16 (1.61 to 2.88)**		**2.04 (1.49 to 2.79)**
	Parent figure vapes			**1.60 (1.19 to 2.16)**	1.20 (0.87 to 1.67)
Vaping susceptibility (n = 1925)	FAS	0.96 (0.90 to 1.03)	1.03 (0.96 to 1.10)	0.98 (0.91 to 1.05)	1.03 (0.95 to 1.11)
	Parent figure smokes		**2.61 (2.07 to 3.29)**		**2.01 (1.54 to 2.62)**
	Parent figure vapes			**2.90 (2.27 to 3.70)**	**2.24 (1.69 to 2.98)**
Perceived smoking norms					
Most adults smoke (n = 1958)	FAS	**0.90 (0.86 to 0.95)**	0.98 (0.92 to 1.04)	**0.92 (0.87 to 0.97)**	0.98 (0.92 to 1.03)
	Parent figure smokes		**2.36 (1.91 to 2.93)**		**2.24 (1.77 to 2.83)**
	Parent figure vapes			**1.62 (1.27 to 2.06)**	1.22 (0.94 to 1.58)
Most adults vape (n = 1901)	FAS	**0.94 (0.90 to 0.99)**	1.00 (0.94 to 1.05)	0.96 (0.91 to 1.01)	1.00 (0.95 to 1.06)
	Parent figure smokes		**1.84 (1.46 to 2.33)**		**1.59 (1.23 to 2.05)**
	Parent figure vapes			**1.95 (1.48 to 2.58)**	**1.67 (1.24 to 2.24)**
More than hardly any children my age smoke (n = 1933)	FAS	0.97 (0.92 to 1.02)	1.00 (0.95 to 1.06)	0.98 (0.92 to 1.03)	1.00 (0.95 to 1.06)
	Parent figure smokes		**1.43 (1.12 to 1.82)**		1.37 (1.09 to 1.72)
	Parent figure vapes			**1.40 (1.10 to 1.78)**	1.27 (1.00 to 1.61)
More than hardly any children my age vape (n = 1896)	FAS	0.97 (0.92 to 1.03)	1.00 (0.95 to 1.06)	0.99 (0.93 to 1.04)	1.00 (0.95 to 1.06)
	Parent figure smokes		**1.53 (1.23 to 1.91)**		**1.33 (1.05 to 1.70)**
	Parent figure vapes			**1.71 (1.37 to 2.14)**	**1.58 (1.22 to 2.04)**
Why adults use e-cigarettes (among those who had heard of e-cigarettes; n = 1847)					
To stop smoking (n = 1677)	FAS	**0.94 (0.88 to 0.99)**	0.99 (0.93 to 1.05)	0.97 (0.92 to 1.02)	1.00 (0.95 to 1.06)
	Parent figure smokes		**2.07 (1.66 to 2.59)**		**1.63 (1.28 to 2.09)**
	Parent figure vapes			**3.63 (2.57 to 5.11)**	**3.20 (2.27 to 4.52)**
To use where smoking is not allowed (n = 1679)	FAS	**1.10 (1.03 to 1.17)**	1.06 (0.99 to 1.13)	1.06 (0.99 to 1.13)	1.03 (0.97 to 1.11)
	Parent figure smokes		**0.61 (0.45 to 0.83)**		0.77 (0.56 to 1.06)
	Parent figure vapes			**0.37 (0.27 to 0.51)**	**0.42 (0.30 to 0.60)**
Cheaper than smoking (n = 1678)	FAS	**1.10 (1.03 to 1.18)**	1.07 (0.99 to 1.16)	**1.08 (1.01 to 1.16)**	1.07 (0.99 to 1.15)
	Parent figure smokes		**0.70 (0.54 to 0.91)**		0.82 (0.63 to 1.06)
	Parent figure vapes			**0.48 (0.34 to 0.67)**	**0.52 (0.37 to 0.73)**

Ns relate to final fully adjusted models. FAS, Family Affluence Scale. Bold indicates *p* < 0.05

**Table 4:** Multivariable logistic regression models examining the association of children’s perceptions of why adults use e-cigarettes with perceived smoking norms, and susceptibility for future smoking and vaping (among children who had heard of e-cigarettes).

	To Quit Smoking	To Use in Public When Smoking is Banned	Because They Are Cheaper than Tobacco
Most adults smoke (n = 1641)	0.92 (0.71 to 1.20)	1.11 (0.85 to 1.47)	1.24 (0.88 to 1.74)
More than hardly any children my age smoke (n = 1623)	1.06 (0.83 to 1.35)	0.92 (0.67 to 1.25)	**1.39 (1.06 to 1.82)**
Smoking susceptibility (n = 1629)	**0.57 (0.41 to 0.79)**	1.21 (0.81 to 1.80)	0.99 (0.62 to 1.59)
Vaping susceptibility (n = 1621)	**0.78 (0.62 to 0.99)**	1.10 (0.78 to 1.55)	1.00 (0.68 to 1.47)

All models adjusted for FAS, sex and parental smoking and vaping. Bold indicates *p* < 0.05

## References

[1] Leonardi-Bee J., Jere M.L., Britton J. (2011). Exposure to parental and sibling smoking and the risk of smoking uptake in childhood and adolescence: A systematic review and meta-analysis. Thorax.

[2] Hallingberg B., Maynard O.M., Bauld L., Brown R., Gray L., Lowthian E., MacKintosh A.-M., Moore L., Munafo M.R., Moore G. (2019). Have e-cigarettes renormalised or displaced youth smoking? Results of a segmented regression analysis of repeated cross sectional survey data in England, Scotland and Wales. Tob. Control.

[3] Moore G.F., Evans R.E., Hawkins J., Littlecott H., Melendez-Torres G., Bonell C., Murphy S. (2019). From complex social interventions to interventions in complex social systems: Future directions and unresolved questions for intervention development and evaluation. Evaluation.

[4] Moore G., Moore L., Littlecott H.J., Ahmed N., Lewis S., Sully G., Jones E., Holliday J. (2015). Prevalence of smoking restrictions and child exposure to secondhand smoke in cars and homes: a repeated cross-sectional survey of children aged 10-11 years in Wales. BMJ Open.

[5] Doku D.T., Acacio-Claro P.J., Koivusilta L., Rimpelä A. (2019). Social determinants of adolescent smoking over three generations. Scand. J. Public Health.

[6] Gregoraci G., Van Lenthe F., Artnik B., Bopp M., Deboosere P., Kovács K., Looman C., Martikainen P., Menvielle G., Peters F. (2017). Contribution of smoking to socioeconomic inequalities in mortality: A study of 14 European countries, 1990–2004. Tob. Control.

[7] West R., Beard E., Brown J. (2018). Trends in Electronic Cigarette Use in England.

[8] Welsh Government (2019). National Survey for Wales 2018–2019: Adult Smoking and E-Cigarette Use.

[9] Beard E., West R., Michie S., Brown J. (2019). Association of prevalence of electronic cigarette use with smoking cessation and cigarette consumption in England: A time series analysis between 2006 and 2017. Addiction.

[10] Hajek P., Phillips-Waller A., Przulj D., Pesola F., Myers Smith K., Bisal N., Li J., Parrott S., Sasieni P., Dawkins L. (2019). A randomized trial of e-cigarettes versus nicotine-replacement therapy. N. Engl. J. Med..

[11] Worku D., Worku E. (2019). A narrative review evaluating the safety and efficacy of e-cigarettes as a newly marketed smoking cessation tool. SAGE Open Med..

[12] Romijnders K., van Osch L., de Vries H., Talhout R. (2018). Perceptions and Reasons Regarding E-Cigarette Use among Users and Non-Users: A Narrative Literature Review. Int. J. Environ. Res. Public Health.

[13] Stepney M., Aveyard P., Begh R. (2019). GPs’ and nurses’ perceptions of electronic cigarettes in England: A qualitative interview study. Br. J. Gen. Pract..

[14] Hammond D. (2019). Outbreak of pulmonary diseases linked to vaping. BMJ.

[15] Alexander L.E.C., Perez M.F. (2019). Identifying, tracking, and treating lung injury associated with e-cigarettes or vaping. Lancet.

[16] Nyakutsikwa B., Britton J., Bogdanovica I., Langley T. (2019). Vitamin E acetate is not present in licit e-cigarette products available on the UK market. Addiction.

[17] George J., Hussain M., Vadiveloo T., Ireland S., Hopkinson P., Struthers A.D., Donnan P.T., Khan F., Lang C.C. (2019). Cardiovascular effects of switching from tobacco cigarettes to electronic cigarettes. J. Am. Coll. Cardiol..

[18] Bullen C., Howe C., Laugesen M., McRobbie H., Parag V., Williman J., Walker N. (2013). Electronic cigarettes for smoking cessation: A randomised controlled trial. Lancet.

[19] West R., Shahab L., Brown J. (2016). Estimating the population impact of e-cigarettes on smoking cessation in England. Addiction.

[20] McNeill A., Brose L., Calder R., Hitchman S., Hajek P., McRobbie H. (2015). E-cigarettes: An evidence update. A report commissioned by Public Health England. Public Health Engl..

[21] The Lancet (2019). E-cigarettes: Time to realign our approach?. Lancet.

[22] Newton J.N. (2019). Time for The Lancet to realign with the evidence on e-cigarettes?. Lancet.

[23] Chapman S., Bareham D., Maziak W. (2018). The Gateway Effect of e-cigarettes; Reflections on main Criticisms. Nicotine Tob. Res..

[24] Conner M., Grogan S., Simms-Ellis R., Flett K., Sykes-Muskett B., Cowap L., Lawton R., Armitage C.J., Meads D., Torgerson C. (2018). Do electronic cigarettes increase cigarette smoking in UK adolescents? Evidence from a 12-month prospective study. Tob. Control.

[25] Voigt K. (2015). Smoking norms and the regulation of e-cigarettes. Am. J. Public Health.

[26] Thompson A. (2019). Vaping is NOT a gateway to cigarettes: Scientists find ‘little evidence vaping normalises smoking among teenagers’. Daily Mail.

[27] Official Journal of the European Union (2014). Directive 2014/40/EU of the European Parliament and of the Council of 3 April 2014 on the Approximation of the Laws, Regulations and Administrative Provisions of the Member States Concerning the Manufacture.

[28] Welsh Government (2015). Public Health (Wales) Bill.

[29] BBC News (2019). India e-cigarettes: Ban announced to prevent youth ‘epidemic’. BBC News.

[30] Doward J., Fraser T. (2019). UK attacked for defence of flavoured e-cigarettes. The Guardian.

[31] Buckell J., Marti J., Sindelar J.L. (2019). Should flavours be banned in cigarettes and e-cigarettes? Evidence on adult smokers and recent quitters from a discrete choice experiment. Tob. Control.

[32] Glasser A., Abudayyeh H., Cantrell J., Niaura R. (2018). Patterns of E-Cigarette Use Among Youth and Young Adults: Review of the Impact of E-Cigarettes on Cigarette Smoking. Nicotine Tob. Res..

[33] East K., Hitchman S.C., Bakolis I., Williams S., Cheeseman H., Arnott D., McNeill A. (2018). The Association Between Smoking and Electronic Cigarette Use in a Cohort of Young People. J. Adolesc. Health.

[34] Levy D.T., Warner K.E., Cummings K.M., Hammond D., Kuo C., Fong G.T., Thrasher J.F., Goniewicz M.L., Borland R. (2018). Examining the relationship of vaping to smoking initiation among US youth and young adults: A reality check. Tob. Control.

[35] Hammond D., Reid J.L., Rynard V.L., Fong G.T., Cummings K.M., McNeill A., Hitchman S., Thrasher J.F., Goniewicz M.L., Bansal-Travers M. (2019). Prevalence of vaping and smoking among adolescents in Canada, England, and the United States: Repeat national cross sectional surveys. BMJ.

[36] Hewitt G., Anthony R., Moore G., Melendez-Torres G.J., Murphy S. (2019). Student Health and Wellbeing in Wales: Report of the 2017/18 Health Behaviour in School-Aged Children Survey and School Health Research Network Student Health and Wellbeing Survey.

[37] Faletau J., Glover M., Nosa V., Pienaar F. (2013). Looks like smoking, is it smoking? Children’s perceptions of cigarette-like nicotine delivery systems, smoking and cessation. Harm Reduct. J..

[38] Been J.V., Millett C., Lee J.T., van Schayck C.P., Sheikh A. (2015). Smoke-free legislation and childhood hospitalisations for respiratory tract infections. Eur. Respir. J..

[39] Faber T., Been J.V., Reiss I.K., Mackenbach J.P., Sheikh A. (2016). Smoke-free legislation and child health. NPJ Prim. Care Respir. Med..

[40] Patel M., Thai C.L., Meng Y.-Y., Kuo T., Zheng H., Dietsch B., McCarthy W.J. (2018). Smoke-free car legislation and student exposure to smoking. Pediatrics.

[41] Filippidis F.T., Laverty A.A., Gerovasili V., Vardavas C.I. (2017). Two-year trends and predictors of e-cigarette use in 27 European Union member states. Tob. Control.

[42] Moore G.F., Littlecott H.J., Moore L., Ahmed N., Holliday J. (2016). E-cigarette use and intentions to smoke among 10-11-year-old never-smokers in Wales. Tob. Control.

[43] Porcellato L., Ross-Houle K., Quigg Z., Harris J., Bigland C., Bates R., Timpson H., Gee I., Bishop J., Gould A. (2018). Is It All Smoke without Fire? Welsh Primary School Children’s Perceptions of Electronic Cigarettes.

[44] Sæbø G., Scheffels J. (2017). Assessing notions of denormalization and renormalization of smoking in light of e-cigarette regulation. Int. J. Drug Policy.

[45] Brown R., Bauld L., de Lacy E., Hallingberg B., Maynard O., McKell J., Moore L., Moore G. (2020). A qualitative study of e-cigarette emergence and the potential for renormalisation of smoking in UK youth. Int. J. Drug Policy.

[46] Torsheim T., Cavallo F., Levin K.A., Schnohr C., Mazur J., Niclasen B., Currie C., FAS Development Study Group (2016). Psychometric validation of the revised family affluence scale: A latent variable approach. Child Indic. Res..

[47] Moore G., Hewitt G., Evans J., Littlecott H.J., Holliday J., Ahmed N., Moore L., Murphy S., Fletcher A. (2015). Electronic-cigarette use among young people in Wales: Evidence from two cross-sectional surveys. BMJ Open.

[48] De Lacy E., Fletcher A., Hewitt G., Murphy S., Moore G. (2017). Cross-sectional study examining the prevalence, correlates and sequencing of electronic cigarette and tobacco use among 11–16-year olds in schools in Wales. BMJ Open.

[49] Kavuluru R., Han S., Hahn E.J. (2019). On the popularity of the USB flash drive-shaped electronic cigarette Juul. Tob. Control.

[50] Hartwell G., Thomas S., Egan M., Gilmore A., Petticrew M. (2017). E-cigarettes and equity: A systematic review of differences in awareness and use between sociodemographic groups. Tob. Control.

[51] Lucherini M., Hill S., Smith K. (2019). Potential for non-combustible nicotine products to reduce socioeconomic inequalities in smoking: A systematic review and synthesis of best available evidence. BMC Public Health.

[52] Thirlway F. (2019). Nicotine addiction as a moral problem: Barriers to e-cigarette use for smoking cessation in two working-class areas in Northern England. Soc. Sci. Med..

